# Diarrheal morbidity and predisposing factors among children under 5 years of age in rural East Ethiopia

**DOI:** 10.1186/s41182-020-00253-4

**Published:** 2020-08-06

**Authors:** Ephrem Tefera Solomon, Sirak Robele Gari, Helmut Kloos, Bezatu Mengistie

**Affiliations:** 1 Ethiopian Institute of Water Resources, Addis Ababa University, Addis Ababa, Ethiopia; 2 College of Health and Medical Sciences, Haramaya University, Harar, Ethiopia; 3 San Francisco Medical Center, University of California, San Francisco, CA USA

**Keywords:** Diarrheal morbidity, Predisposing factors, Children under 5 years of age, Handwashing, Dire Dawa

## Abstract

**Background:**

Diarrheal diseases remain a leading cause of preventable death among children under-five in low- and middle-income countries (LMICs). In Ethiopia, diarrhea is the major contributor to deaths for children under the age of 5 years. In order to develop prevention strategies for the alleviation of childhood diarrhea, it is necessary to identify the important predisposing factors. These predisposing factors have been observed to vary by location across Eastern Ethiopia. Moreover, the evidence on prevalence and determinants of diarrhea among children under 5 years of age in Dire Dawa and its suburbs is very limited and those available have been erratic. The objective of this study was to determine the prevalence and predisposing factors of diarrhea among children under the age of 5 years in rural Dire Dawa, East Ethiopia.

**Methods:**

A community-based cross-sectional study was conducted in rural Dire Dawa City Administration in May 2018. Multistage sampling technique was employed to recruit 1180 under-five children from the rural population of Dire Dawa City Administration. Data on socio-demographic, environmental, and child hygiene-related factors were collected by trained data collectors using a structured questionnaire. Logistic regression was used to identify independent risk factors for childhood diarrhea.

**Results:**

The 2-week prevalence of diarrhea among the under-five children was 23% (95% CI 20.8–25.7%). Maternal diarrhea (AOR = 2.22, 95% CI 1.10–4.47), handwashing after contact with child feces (AOR = 6.27, 95% CI 2.01–19.55), use of a dipper to draw water from containers (AOR = 2.88, 95% CI 1.41–5.89), and presence of a refuse disposal facility (AOR = 2.47, 95% CI 1.09–5.60) were the significant predisposing factors of diarrhea.

**Conclusion:**

Our study identified a high burden of childhood diarrheal disease in rural Dire Dawa City Administration in Eastern Ethiopia. The identified risk factors were maternal diarrhea, handwashing after contact with child feces, use of a dipper to draw water from containers, and presence of refuse disposal facility. To minimize the risk of diarrhea, health education programs focusing on good hygiene practice and sanitation as well as early treatment are recommended.

## Introduction

Diarrheal diseases remain a leading cause of preventable death, especially among children under-five in LMICs [1]. Globally, an estimated 2 billion cases of diarrhea occur each year, and 1.9 million children under the age of 5 years, mostly in LMICs, die from diarrhea [2]. It is defined as the passage of three or more loose or liquid stools per day [3]. Several studies identified the following risk factors for under-five diarrhea: mother’s/guardian’s age, education, and occupation [4, 5]. Similar studies detected predisposing factors such as maternal history of recent diarrhea, lack of latrines, age of the child [6], and absence of refuse disposal facilities [7] in Ethiopia. However, some practices such as use of treated water, handwashing before feeding under-five child, and use of narrow-mouth container for storage of drinking water have been noted to significantly lower the risk of childhood diarrhea [8, 9].

In Ethiopia, diarrhea is the major contributor to the deaths for children under the age of 5 years [10]. According to the 2016 Ethiopia Demographic and Health Survey, the prevalence of under-five diarrhea was 12.1% and 12% in Ethiopia and Dire Dawa, Eastern Ethiopia, respectively [11]. According to the 2017 Dire Dawa City Administration Regional Health Bureau’s Annual Report, the prevalence of under-five diarrhea in the administration was 35.5% (Dire Dawa City Administration Regional Health Bureau: 2017 Facility Information (unpublished observation)). Studies carried out in different localities of Ethiopia revealed high burden of diarrheal morbidity. For instance, 2-week period prevalence was 22.1% in rural areas of North Gondar Zone; 28.9% in Nekemte Town in western Ethiopia; 27.3% in Jigjiga District, Somali Region; and 22.5% in Kersa District both in Eastern Ethiopia [7, 12–14].

Despite the high prevalence of childhood diarrhea in Ethiopia, there is clear variation in the epidemiological reports from different localities. Moreover, there is little information on the prevalence and determinants of diarrhea among children under 5 years of age in Dire Dawa and its suburbs, and existing results are inconsistent. For instance, a systematic review and meta-analysis revealed that the prevalence of under-five diarrhea in Dire Dawa was 26% [15] and in rural *kebeles* of Dire Dawa it was 37.1% [16]. Children using water from unprotected springs or wells were more likely to be infected with diarrhea-causing bacteria [17]. The objective of this study is to determine the prevalence and predisposing socio-demographic, behavioral, and environmental factors of diarrhea among children under the age of 5 years in rural districts of Dire Dawa, East Ethiopia.

## Methods

### Study design and area

A community-based cross-sectional study was conducted in rural Dire Dawa City Administration in May 2018. Dire Dawa is the second largest city in Ethiopia located about 505 kilometers to the east of Addis Ababa (Dire Dawa Administration Statistical Abstract 2013/14–2014/15; Prepared by Dire Dawa Administration Bureau of Finance and Economic Development [unpublished]). The City administration has nine urban and 38 rural *kebeles* (neighborhood associations). There are two government hospitals, five private hospitals, 15 health centers, and 33 health posts (Dire Dawa administration Regional Health Bureau: 2017 six months report [unpublished]). According to the 2007 National Census Report, the total population of Dire Dawa City Administration was 341,834, of which just over half (50.2%) were males and 31.8% lived in rural areas (Dire Dawa Administration Statistical Abstract 2013/14–2014/15; prepared by Dire Dawa Administration Bureau of Finance and Economic Development [unpublished]). According to the Regional Health Bureau Report of 2017, there were 34,150 households in the four districts of rural Dire Dawa with 20,118 children under the age of 5 years (Dire Dawa Administration Regional Health Bureau: 2017 Facility Information and Document review from Dire Dawa Administration Disaster Risk Management Bureau [Unpublished]).

### Inclusion and exclusion criteria

Households having one child aged between 6 and 59 months were included. All, under-five children with persistent diarrhea and caretakers or household heads who were severely ill and unable to respond to the questionnaire were excluded.

### Population and sampling

All households with at least one under-five child in the 38 rural *kebeles* (a *kebele* is the smallest administrative unit of Ethiopia, similar to a ward, a neighborhood) of the four districts were the source population whereas the under-five children in the randomly selected four *kebeles* were the study populations. The sample size was calculated using the formula for estimation of a single population proportion [18], *n* = *z*^2^_1−*α*/2_*P*(1 − *P*)/*d*^2^, where *z*_1−*α*/2_ is 1.96; *P* is prevalence of diarrhea among children under 5 years of age in Eastern Ethiopia, Kersa District, estimated to be 22.5% [14]; *d* is acceptable margin of error is 5% (0.05), and the level of confidence is 95%. The calculated sample size was 268. After considering a design effect of four accounting for two stage sampling [19] and 10% compensation for expected non-response, the sample size became 1180. In this study, four of 38 *kebeles* were selected using simple random sampling, and the participating households were also selected using simple random sampling from a sampling frame known as “family folders” in the health centers and health posts. Accordingly, a design effect of four is introduced as an adjustment for the multi-stage sampling employed.

A multi-stage sampling procedure was used to recruit the calculated sample size from the rural population of Dire Dawa City Administration. Four of the 38 rural *kebeles* were selected using a simple random sampling technique. The *kebeles* in the four districts were listed and written on pieces of papers; then, four *kebeles* were randomly selected using a draw method. Those eligible for this study were households having at least one under-five child. Then, the study households were selected again using a simple random sampling technique from the “family folders” that were regularly updated by the respective health centers and health posts. In households with more than one under-five child, the index under-five child was selected by using the lottery method.

### Data collection

All data were collected using a structured questionnaire that was administered to the study participants by trained data collectors. The questionnaire was first prepared in English and then translated into the local language (Afaan Oromo). It was then back translated to English by an anonymous translator to ensure the consistency of the two versions. Sixteen data collectors and four supervisors were employed. The data collectors were local residents of the respective *kebeles* who completed grade 10 and who spoke the local language, and the supervisors were also local residents who completed grade 12. All data collectors and supervisors received two days of training on interviewing techniques and data collection from the first author. Information on diarrhea for the included study participant in the household in the 2 weeks prior to the interviews and information related to diarrhea was collected from the mother or guardian of each under-five child.

### Study variables

#### Dependent variable

Childhood diarrhea in the 2 weeks preceding the survey was the dependent variable.

#### Independent variables

Child age [5] and sex [20], mother’s age and education [4], mothers occupation [5], father’s education [21], number of under-five children [22], quality of the main floor material [5], drinking water source [23], maternal diarrhea [6], storing water in containers without lids [20], use of dippers to draw water from water storage containers [9], availability of a latrine [23], method of under-five child stool disposal [23], improper refuse disposal [24], availability of handwashing (handwashing is defined as the act of cleaning one’s hands to remove soil, grease, microorganisms, or other unwanted substances) facility [14], handwashing before preparing food and before eating food [8], before feeding a child [13], handwashing after eating and after contact with child feces [13], visible feces in the compound [7], and visible feces on the latrine slab [7] were the independent variables.

### Data quality assurance

Adequate training was given to data collectors and supervisors on techniques of interviewing and general approaches to community motivation and supervision. The data collection tool was pretested in a nearby *kebele* that was not included in the study in order to distinguish language barriers and contextual differences. Questionnaires were checked daily for their consistencies and completeness by the supervisors. The questionnaires were checked for uniformity of units of measurement used as well as for missing data that were obtained the next day by data collectors. Supervisors also checked the accuracy of data by randomly interviewing some of the participating households. At the end of data collection, the principal investigator verified proper data collection each day by holding a short meeting with the supervisors.

### Data analysis

The collected data were cleaned and entered onto EPI-Data Version 3.1 and analyzed using analysis format, SPSS for Windows Version 23. Descriptive statistics such as percentages, means, standard deviations, and proportions of the study variables were carried out. Explanatory variables were tested for multi-collinearity using the variance inflation factor (VIF) and the tolerance test.

The explanatory variables were classified into three major categories: socio-demographic, environmental, and child- and hygiene-related factors. Bivariate analysis was used to examine the association between each of the three explanatory variables and the outcome variable, diarrhea. Then, multivariable logistic regression was used to identify independent risk factors of childhood diarrhea after incorporating explanatory variables scoring a *p* value of less than 0.1 in bivariate regression; despite the limitations of the logistic regression such as the assumption of linearity between the dependent variable and the independent variables, in the real world, the data is rarely linearly separable. If the number of observations are less than the number of features, logistic regression should not be used; otherwise, it may lead to overfit and logistic regression can only be used to predict discrete functions. *P* values less than 0.05 were considered significant in the multivariable regression.

## Results

A total of 1146 households included in this study with a response rate of 97.1%. Out of the 1146 under-five children, 576 (50.3%) were males. More than three-quarters, 77.8%, were from the Oromo ethnic group and 22.2% were Somali. Socio-demographic characteristics of the parents of the children are shown in Table 1. The 2 week prevalence of diarrhea in rural Dire Dawa among the under-five children was 23% (95% CI 20.8–25.7%).

**Table 1:** Socio-demographic characteristics of the parents of the under-five children, rural areas of Dire Dawa City Administration, 2018

Variables			Frequencies	Percentages
**Mothers’/guardians’**	**Ethnicity**	Oromo	926	80.8
		Somali	220	19.2
	**Religion**	Muslim	1141	99.6
		Orthodox Christian	4	0.3
		Protestant	1	0.1
	**Educational status**	No formal education	789	68.8
		Primary education	298	26.0
		Secondary education	44	3.8
		Above secondary education	15	1.3
	**Occupation**	Housewife	843	73.6
		Farmer	261	22.8
		Merchant	21	1.8
		Day laborer	3	0.3
		Government employee	18	1.6
**Fathers’**	**Ethnicity**	Oromo	841	73.4
		Somali	244	21.3
	**Religion**	Muslim	1084	94.6
		Orthodox Christian	1	0.1
	**Educational status**	No formal education	576	50.3
		Primary education	371	32.4
		Secondary education	88	7.7
		Above secondary education	50	4.4
	**Occupation**	Farmer	961	83.9
		Merchant	20	1.7
		Day laborer	16	1.4
		Government employee	88	7.7

### Socio-demographic factors

The majority of the mothers/guardians had no formal education ((crude odds ratio) OR = 3.19, 95% CI 1.26–8.10), were single (OR = 1.10, 95% CI 0.60–1.99), and were housewives (OR = 0.97, 95% CI 0.71–1.32). Nearly half of the households had a single room (OR = 1.58, 95% CI 1.20–2.09). Mothers’/guardians’ educational status and number of living rooms had a statistically significant effect on under-five diarrhea. Socio-demographic factors of diarrhea are indicated in Table 2.

**Table 2:** Distribution of diarrheal disease in relation to socio-demographic factors, rural Dire Dawa, Eastern Ethiopia, 2018

Factors	Diarrhea (***n*** = 1146)		Crude OR^a^ (95% CI)^b^
	Yes	No	
**Age of the mother/guardian (years)**			
18–24	38	111	1.14 (0.71–1.84)
25–34	172	591	0.97 (0.69–1.37)
≥ 35	54	180	1
**Education of mother/guardian**			
No formal education	180	609	3.19 (1.26–8.10)
Primary education	79	219	3.90 (1.50–10.09)
Secondary and above	5	54	1
**Occupation of the mother/guardian**			
Housewife	193	650	0.97 (0.71–1.32)
Miscellaneous	71	232	1
**Age of the father (years)**			
20–39	188	630	1.01 (0.73–1.40)
≥ 40	61	206	1
**Education of the father**			
No formal education	143	433	1.26 (0.94–1.67)
Primary and above	106	403	1
**Family size**			
Five or less	182	592	1
Six or above	82	290	0.92 (0.68–1.24)
**Living with spouse**			
Yes	249	836	1
No	15	46	1.10 (0.60–1.99)
**Number of living rooms**			
One	152	407	1.58 (1.20–2.09)
Two and above	112	475	1

^a^Odds ratio

^b^95% confidence interval

### Child- and hygiene-related factors

Analysis of child- and hygiene-related factors showed that most of the under-five children practiced open defecation (OR = 1.42, 95% CI 1.06–1.89) and some of the mothers or guardians had diarrhea 2 weeks before the interview (OR = 3.83, 95% CI 2.88–5.14). Few mothers/guardians washed their hands with soap before eating food (OR = 1.74, 95% CI 1.03–2.93), and most of the mothers/guardians did not wash their hands with soap after cleaning children’s bottom (OR = 1.83, 95% CI 1.19–2.80). Open defecation, maternal diarrhea, and mother’s/guardian’s handwashing habit before eating and after cleaning children’s bottom were significantly associated with childhood diarrhea (Table 3).

**Table 3:** Diarrheal disease in relation to child- and hygiene-related factors, rural Dire Dawa, Eastern Ethiopia, 2018

Factors	Diarrhea (***n*** = 1146)		Crude OR (95% CI)
	Yes	No	
**Gender**			
Male	128	448	1
Female	136	434	1.10 (0.83–1.44)
**Age**			
6–11	19	62	1.01 (0.59–1.74)
12–23	51	179	0.94 (0.66–1.34)
> 23	194	641	1
**Number of under-five children in the household**			
One	123	445	1
Two and above	141	537	1.17 (0.89–1.54)
**Open defecation by the under-five child**			
No	90	373	1
Yes	174	509	1.42 (1.06–1.89)
**Maternal diarrhea**			
No	128	691	1
Yes	136	191	3.83 (2.88–5.14)
**Availability of handwashing facility**			
No	204	661	1.14 (0.82–1.57)
Yes	60	221	1
**Mother washing hands before preparing food**			
No	184	667	1.23 (0.77–1.97)
Yes	24	107	1
**Mother washing hands before eating food**			
No	194	683	1.74 (1.03–2.93)
Yes	18	110	1
**Mother washing hands before feeding child**			
No	178	644	1.19 (0.79–1.77)
Yes	35	150	1
**Mother washing hands after defecation**			
No	175	631	1.12 (0.76–1.67)
Yes	37	150	1
**Handwashing after washing child’s bottom**			
No	177	592	1.83 (1.19–2.80)
Yes	29	177	1

### Environmental factors

Analysis of environmental factors showed that some households had flowing water daily for 1 h or less (OR = 2.54, 95% CI 1.78–3.63). The majority of the households had a refuse disposal facility (OR = 3.19, 95% CI 2.16–4.72) and disposed of the stools of under-five children in and around the compound (OR = 1.91, 95% CI 1.35–2.71). The length of daily supply of water from the public tap, use of a dipper to draw water from containers, presence of a refuse disposal facility, stool disposing method for under-five children, presence of feces, and refuse in and around the house were significantly associated with childhood diarrhea (Table 4).

**Table 4:** Distribution of diarrheal disease in relation to environmental factors, rural Dire Dawa, Eastern Ethiopia, 2018

Factors	Diarrhea (***n*** = 1146)		Crude OR (95% CI)
	Yes	No	
**Source of drinking water**			
Improved	189	662	1
Unimproved	75	220	1.19 (0.88–1.63)
**Daily availability water supply from the public tap**			
≤ 1 h	69	122	2.54 (1.78–3.63)
≥ 2 h	120	540	1
**Use of dipper to draw water from container**			
No	53	476	1
Yes	211	406	4.67 (3.36–6.49)
**Latrine present**			
No	168	522	1.21 (0.91–1.60)
Yes	96	360	1
**Presence of refuse disposal facility**			
No	231	606	3.19 (2.16–4.72)
Yes	33	276	1
**Under-five children stool disposing method**			
Burying and disposing out of the compound	97	385	1
Disposing in and around the compound	78	162	1.91 (1.35–2.71)
**Feces in and around the house**			
No	139	582	1
Yes	125	300	1.75 (1.32–2.31)
**Refuse in and around the house**			
No	133	542	1
Yes	131	340	1.57 (1.20–2.10)

### Predisposing factors of diarrhea among under-five children

In the final multivariable regression, four models were tested to identify the predisposing factors for childhood diarrhea. In the fourth model, four predisposing factors were independently associated with childhood diarrhea. They are maternal diarrhea (AOR (adjusted odds ratio) = 2.22, 95% CI 1.10–4.47), handwashing after contact with child feces (AOR = 6.27, 95% CI 2.01–19.55), use of a dipper to draw water from a container (AOR = 2.88, 95% CI 1.41–5.89), and presence of refuse disposal facility (AOR = 2.47, 95% CI 1.09–5.60).

The odds of diarrhea in children of mothers/guardians who had had diarrhea were 2.22 times higher than children from mothers/ guardians who had had not diarrhea. Children from mothers/guardians who had not washed their hands with soap after contact with child feces had 6.27 times higher odds of diarrhea than their counter parts. Similarly, children from households using a dipper to draw water had 2.88 higher odds of diarrhea than children from households not using a dipper. Likewise, children in households without a refuse disposal facility had 2.47-folds of acquiring diarrhea than children in households with a refuse disposal facility (Table 5).

**Table 5:** Multivariable regression predicting the probability of diarrhea among under-fives, rural Dire Dawa, Eastern Ethiopia, 2018

Predisposing factors	Model I, AOR* (95% CI)	Model II, AOR (95% CI)	Model III, AOR (95% CI)	Model IV, AOR (95% CI)
**Educational status of mother/guardian**				
No formal education	2.89 (1.13–7.35)^**^			2.92 (0.28–30.10)
Primary education	3.76 (1.49–9.76)^**^			3.42 (0.33–35.84)
Secondary and above	1			1
**Number of living rooms**				
Single room	1.59 (1.20–2.10)^**^			0.84 (0.45–1.57)
Two or above	1			1
**Open defecation practice by the U5 child**				
No		1		1
Yes		1.52 (1.07–2.15)^**^		1.30 (0.65–2.63)
**Maternal diarrhea**				
No		1		1
Yes		3.30 (2.33–4.66)^**^		2.22 (1.10–4.47)^**^
**Mother’s handwashing before eating food**				
No		1.22 (0.64–2.34)		0.77 (0.23–2.64)
Yes		1		1
**Handwashing after contact with child feces**				
No		1.69 (1.03–2.77)^**^		6.27 (2.01–19.55)^**^
Yes		1		1
**Stay time of water supply from the public tap**				
< 1 h			1.83 (1.09–3.06)^**^	1.14 (0.47–2.77)
> 2 h			1	1
**Use of dipper to draw water from container**				
No			1	1
Yes			3.01 (1.73–5.23)^**^	2.88 (1.41–5.89)^**^
**Presence of refuse disposal facility**				
No			3.31 (1.69–6.51)^**^	2.47 (1.09–5.60)^**^
Yes			1	1
**Under-five children stool disposing method**				
Burying and disposing outside the compound			1	1
Disposing in and around the compound			1.86 (1.16–2.99)^**^	0.83 (0.40–1.73)
**Presence of feces in or around the house**				
No			1	1
Yes			2.43 (1.30–4.52)^**^	1.70 (0.76–3.82)
**Presence of refuse in or around the house**				
No			1	1
Yes			0.57 (0.29–1.09)	0.83 (0.35–1.97)

*Adjusted odds ratio

**p < 0.05

## Discussion

In this study, we explored the socio-demographic, environmental, and child- and hygiene-related predisposing factors of diarrhea in children younger than 5 years of age in rural areas of Dire Dawa City Administration. Recent history of maternal diarrhea, absence of handwashing after contact with child feces, use of a dipper for drawing water from the container, and non-availability of refuse disposal facilities were the significant predisposing factors of under-five diarrhea.

The 2-week prevalence of under-five diarrhea was 23% (95% CI 20.8%, 25.7%). Similar rates were reported by other studies in Ethiopia; for example, 22.1% in North Gondar Zone [12], 22.1% in Benishangul Gumuz Region [22], 27.3% in Jigjiga District [13], and 22.5% in Kersa District [14]. Similar studies in other African countries revealed comparable results. For example, in Egypt, diarrhea prevalence was 23.6% [21], in Cameroon 23.8% [9], and in Ghana 19.2% [25]. However, the rate was markedly higher than those reported by studies in Farta *Wereda* in northwest Ethiopia (16.9%) [26], Mecha District in West Gojam Zone (18%) [6], and at the national level (12.0%) [11]. But, were lower than those studies conducted in the country, Nekemte Town in western Ethiopia (28.9%) [7] and Arba-Minch District in southern Ethiopia (30.5%) [27]. These differences may be due to variations in socio-economic, behavioral, and environmental factors.

Mothers’/guardians’ diarrhea is one of the risk factors of diarrhea in under-five children. In our study, after adjusting for all predictors of childhood diarrhea, under-five children from mothers/guardians who had diarrhea recently were two times more likely to develop diarrheal disease than under-five child from mothers/guardians who did not have diarrhea recently (adjusted OR = 2.22 and 95% C.I. is 1.10–4.47). This finding corroborates studies in Jigjiga District, Somali Region [13]; in Mecha District, West Gojam Zone [6]; in rural areas of Shebedino District [28]; and in Medebay Zana District, northwest Tigray [29]. These studies indicate that mother/guardian-child transmission of diarrhea may occur via contaminated food and water and mothers’/guardians’ lack of awareness of these diarrhea risks.

The five critical times for handwashing with soap and water were before preparing food, before eating food, before feeding under-five child, after defecation, and after contact with feces of an under-five child [30]. Accordingly, mothers’/guardians’ unhygienic handwashing practices after cleaning children is another exogenous factor for the child to acquire the disease. In this study, after adjusting for all predictors of childhood diarrhea, children of mothers/guardians who did not wash their hands using soap after contact with child feces were six times more likely to develop diarrheal disease than their counter parts. This finding is similar to reports from Arba-Minch District [27]; Sheko District in southwest Ethiopia [24]; Jigjiga District [13]; Banten Province, Indonesia [31]; and southwest Nigeria [32]. The possible explanation could be that mothers/guardians failing to wash their hands after contact with child feces are exposing their children to diarrheal pathogens.

Water may become unsafe at any point between collection and use. Clean water can easily become contaminated when dirty cups are used [33]. In the current study, after controlling for all confounders of under-five diarrhea, children of mothers or guardians who used dippers to draw water from the container were three times more likely to develop diarrheal disease than their counterparts who did not use dippers. The same relationship between the size of openings of water jars and diarrhea in children was reported in Cameroon [9] and Malaysia [34]. This could be explained by the introduction of pathogens into water stored especially in wide-mouthed jars through contaminated hands as well as dirty dippers. This finding was corroborated by a study in Zambia [35].

This study associated the presence of trash and flies with diarrhea, corroborating other studies [33]. After adjusting for all predictors of childhood diarrhea, under-five children from households that did not have a refuse disposal facility were two times more likely to develop diarrhea than children from households that had these facilities, which is consistent with studies in Nekemte Town, Western Ethiopia [7], and in Sheko District, Southwest Ethiopia [24]. This may be due to increased risk of disease transmission around refuse dumps.

### Strengths and limitations

The current study had several strengths and limitations. Being a community-based type of study and the use of multiple documents to prepare the questionnaire are the strengths of this study. However, the use of cross-sectional study design method is a limitation because causal inferences are very difficult to address. The time when the study was conducted could be another limitation of this study because the study was conducted in May, the driest and the hottest month of 2018. Therefore, future studies addressing all seasons of the year are encouraged. We collected information on diarrhea for the 2 weeks prior to the interview, which might have caused recall bias. However, this was minimized by asking mothers/caregivers repeatedly about the specific days when children had diarrhea. The other limitation was that data were collected by field workers and supervisors who are not paramedical staff. However, the field workers and supervisors were properly trained on data collection and supervision, respectively.

## Conclusion and recommendations

Nearly one out of four children under the age of 5 years was found to have diarrhea. Despite efforts made by different stakeholder to alleviate the health problem related with childhood diarrhea, this public health problem needs further investigation to identify the possible predisposing factors and to plan and implement prevention strategies. Predisposing factors of maternal diarrhea, poor handwashing practices by the mothers/guardians after cleaning the children’s bottoms, use of scoops for drawing water from the container, and absence of refuse disposal facilities were significantly associated with diarrhea.

Based on the findings of this study, the authors recommend educating mothers and guardians regarding the mode of transmission of diarrheal diseases and how to protect themselves and their under-five children including early treatment as preventive measures. The authors also recommend that mothers and guardians be trained in proper handwashing specifically after cleaning their children’s bottom, safe storage, and handling of drinking water after bringing it from the source and on proper disposing of household wastes to minimize exposure to diarrheal pathogens among their children. In the long term, the situation may be improved by providing formal education above primary level to the rural population; specifically to mothers/caregivers to make them aware the risk and prevention of diarrhea.

## Data Availability

Data on which the conclusions of the manuscript rely are summarized in texts and tables in this manuscript. Nevertheless, if need be the main data in machine readable format can be obtained from the principal author (ETS) using formal ways of communication.

## Abbreviations

AOR: Adjusted odds ratio
OR: Odds ratio
CI: Confidence interval
